# Nephrolithiasis Caused by Ceftriaxone in a 3-Year-Old Child with Ureteropelvic Junction Obstruction

**DOI:** 10.1155/2009/365962

**Published:** 2009-05-26

**Authors:** Vesna Stojanovic, Gordana Djuric Vijatov

**Affiliations:** ^1^Department of Nephrology, Institute for Child and Youth Health Care of Vojvodina, Hajduk Veljka 10, 21000 Novi Sad, Serbia; ^2^Department of Allergology, Immunology and Reumathology, Institute for Child and Youth Health Care of Vojvodina, Hajduk Veljka 10, 21000 Novi Sad, Serbia

## Abstract

We report the case of a 3-year-old boy with urinary tract malformation (left sided stenosis of the ureteropelvic junction) which was precipitating factor for ensuing nephrolithiasis of the left kidney during the therapy with ceftriaxone. The treatment with spasmolytics was initiated, together with the forced parentheral hydration. After 3 weeks, there was no evidence of calculi in the urinary tract.

## 1. Introduction

Nephrolithiasis is a condition characterized by the formation of crystallized material in the urinary system [1]. The occurrence of urolithiasis is most significantly influenced by metabolic disorders, urinary tract obstruction with urine stasis and urinary tract infection [1–3].

Ceftriaxone is a third generation cephalosporin, the broad spectrum antibiotic with a long plasma half life. It is widely used to treat infections during childhood. Ceftriaxone is primarily eliminated via kidneys (33–67%), with the remainder eliminated via biliary system. Ceftriaxone may bind calcium and form insoluble precipitates leading to biliary pseudolithiasis and nephrolithiasis [4–6].

The authors report the case of a 3-year-old boy with urinary tract malformation (left-sided stenosis of the ureteropelvic junction) who developed nephrolithiasis of the left kidney during ceftriaxone therapy.

## 2. Case Report

A 3-year-old boy was hospitalized in our department with the diagnosis of Henoch-Schönlein purpura (HSP). The symptoms started 12 days before the admittance with fever, throat, and middle ear infection. He was treated with antibiotics (amoxicillin/clavulanic acid). On the third day of therapy, he presented, at first individual, and later generalized purpuric lesions on the lower extremities. He was initially hospitalized in the regional hospital where he was treated with penicillin. The skin lesions continue to spread, with the occurrence of ankle joints edemas as well as the blood-stained mucus stool. The boy was finally referred to our department for further treatment. 

There was no evidence of previous more severe diseases or the diseases of urinary tract. Family history was negative in concern to nephrolithiasis and any other hereditary disease. 

Physical examination confirmed afebrile boy (body weight 15 kg, height 95 cm) with partially generalized palpable purpura on the gluteus and lower extremities. Also, edemas on both ankle joints and feet, as well as of eyelids were present. Deep palpation of the abdomen did not elicit pain. Physical findings of other systems were normal. 

**Figure 1:**
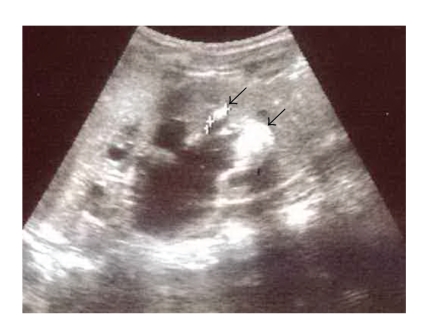
Calculi in the calices of the inferior pole of the left kidney 15.5 mm, and interpolarly sized 6.5 mm

Laboratory analyses revealed positive acute phase reactants and normal basic biochemical and immunologic analyses. The fecal occult blood test in the stool was positive on several occasions. The coproculture was negative. Urine finding was normal. Initial ultrasonography (US) of the abdomen and urinary tract: dimensions of the left kidney-70 × 36 mm, parenchima thickness-9.6 mm, with hydronephrosis-17 × 20 mm. The US findings of the right kidney and other US findings were normal. Immediately upon the admittance, treatment with corticosteroid therapy started-methylprednisolone 1 mg/kg/day. On the third day of treatment the boy was subfebrile; the chest X-ray revealed right pneumonia so the therapy with ceftriaxone 100 mg/kg/day was initiated. The entire time the boy was in a good general condition, afebrile during the further course of the disease, well hydrated. On the 6th day of ceftriaxon treatment, the boy complained of having an intensive pain in the abdomen and vomited several times. Mild tenderness and rigidity in the whole abdomen region was present. The native X-ray of abdomen did not show urolithiasis. US of the abdomen and urinary tract showed left-sided hydronephrosis-38 × 37 mm and presence of calculi in the calices of the inferior pole of the left kidney 15.5 mm, and interpolarly sized 6.5 mm (Figure 1). In the ureteropelvic junction, the calculus sized 6.5 mm was visualized. Other US findings were normal. During the US examination, the calculi were seen to disintegrate into sand which was accumulating in the bladder. The chemical analysis of the calculus has not been performed because of technical problems.

Venous blood gas analysis and the electrolythes level and cystine level in random urine specimen were within normal range. Urine culture was negative. Urine sediment was normal, without hematuria. The ceftriaksone therapy was stopped immediately. The treatment with spasmolytics was initiated, together with the forced parentheral hydration. Following the administered therapy the boy stopped to complain of abdominal pain and had no further symptoms until the end of hospitalisation. The successive US examinations of the urinary tract confirmed the calculi size reduction within the kidney, and 3 weeks following the interrupted ceftriaxone therapy, there were no evidence of calculi in the urinary tract, whereas the initially diagnosed left kidney hydronephrosis persisted. Simultaneously, signs of regression of the basic disease—HSP were noted. In the further course of the disease and during control examinations, the patient underwent the intravenous urography which confirmed the presence of left kidney stenosis of the ureteropelvic junction. 

## 3. Discussion

Clinical studies suggest that ceftriaxone can lead to a reversible precipitation (on US, the calculi present a posterior acoustic shadow) in the gall bladder resembling the cholelithiasis. This complication is called a biliary pseudolithiasis or a reversible cholelithiasis. A prospective study showed biliary pseudolithiasis in 25–45% of patients who were treated with ceftriaxone [4, 5, 7].

Up to now, there were about 10 reports published on ceftriaxone-induced nephrolithiasis. Ceftriaxon is an anion and, when drug concentrations in blood are high, these anions can bind with calcium ions to form insoluble complexes that precipitate in the biliary system. It appears that stones can form in the same way in the renal collecting system [5, 8]. All described calculi were of small dimensions (a few mm), whereas Mohkam have recently reported a case of a 3-year-old child presented with the calculi in both kidneys sized 10 and 15 mm [9]. In most reports, the ceftriaxone-induced nephrolithiasis occurred during 8–10 days of treatment, with the doses of 50 to 100 mg/kg/day. Shortly after the interruption of the ceftriaxone therapy, usually 5 days to 3 weeks, rarely after a longer time, the calculi were eliminated both from the urinary tract and the gall bladder, [8–12].

 In the case of our patient, a complete elimination of the calculi occurred after 3 weeks of the therapy interruption. Some authors reported more difficult elimination of the calculi from the inferior pole of kidney which could be either prolonged or impossible [13]. Our patient had a calculus localized in the lower pole of the left kidney, but nevertheless it has been eliminated very quickly. Perhaps the intensive parentheral hydration associated with the administered spasmolytics (the other authors did not report treatment procedure administered in this protocol) led to the prompt elimination of the calculus from that region of the kidney. 

It is thought that the risk factors leading to ceftriaxone-induced nephrolithiasis comprise a positive family history, high doses of ceftriaxone (over 2 g/day), quick application of the drug, dehydration, together with the administration of nephrotoxic drugs, whereas some authors believe that metabolic disorders, such as hypercalciuria and hypocitraturia could also be metabolic factors favoring crystallization of calcium ceftriaxonate. Essentially, the ceftriaxone-induced urolithiasis is self-limited without long-term complications [1–3, 13, 14].

Our patient received drug doses of 100 mg/kg/day. It is also thought that high doses of the drug can lead to lithiasis, but this problem can also occur at a normal dose levels. Perhaps higher doses of the drug lead to larger size calculi [6]!? 

Mohkam analyzed US findings in 284 children who were receiving ceftriaxone for pyelonephritis. At the end of the treatment, 1.4% of the children were presented with the cefrtiaxone-induced urolithiasis [9]. Biner reported that 0.6% of children treated by ceftriaxone developed urolithiasis and 10% developed biliary pseudolithiasis [15] whereas Avci reported on 7.8% children with ceftriaxone-induced nephrolithiasis [6].

The predisposing factors which might have led to the development of urolithiasis in our patient were urinary tract malformation (left-sided stenosis of the ureteropelvic junction), as well as the corticosteroid therapy (although laboratory analyses did not prove hypercalciuria). In two large studies, Mohkam et al., and Avci et al., did not diagnose urinary tract malformation in patients with detected ceftriaxon-induced nephrolitiasis [6, 9].

Finally, it can be concluded that the treatment with ceftriaxone requires US monitoring of the urinary tract and abdomen as well as the adequate hydration of the child. A special attention has to be paid to the children who have some predisposing factors for the development of urolithiasis.
